# Lesson learned from early and long-term results of 327 cases of coexisting surgical abdominal diseases and aortic aneurysms treated in open and endovascular surgery

**DOI:** 10.1007/s13304-012-0137-4

**Published:** 2012-03-11

**Authors:** Stefano Bonardelli, Edoardo Cervi, Franco Nodari, Cristina Guadrini, Camilla Zanotti, Stefano Maria Giulini

**Affiliations:** 1 Department of Medical and Surgical Sciences, Unit and Chair of Vascular Surgery, Universita` degli Studi, A.O. Spedali Civili Brescia, Brescia, Italy; 2 Department of Medical and Surgical Sciences, Unit and Chair of Clinical Surgery, Universita` degli Studi, A.O. Spedali Civili Brescia, Brescia, Italy; 3 Dipartimento di Scienze Mediche e Chirurgiche, A.O. Spedali Civili, Policlinico Satellite, Piazzale Spedali Civili 1, 25124 Brescia, Italy

**Keywords:** Abdominal aortic aneurysm, General surgery, Associated diseases

## Abstract

Patients with abdominal aortic aneurysm (AAA) frequently have other abdominal pathologies of surgical interest (other diseases, OD). Out of 1,375 elective open aortic replacements for AAA, 315 cases with OD were subdivided in Group 1 (82 patients with “clean wound” OD) and Group 2 (233 patients with “clean-contaminated wound” OD). The results of the sub-groups in which OD was treated at the same time as AAA were analysed (1a, 66 cases and 2a, 86 cases) and compared with OD not treated at the same time as AAA (1b, 16 cases and 2b, 147 cases). EVAR was done in 12 patients with a infrarenal AAA and concomitant abdominal disease. In this group post-operative complications occured in two patients (endoleaks) and no sign of endograft infection was developed. Mean follow-up was 36 months. Mortality was 0% in Group 1a, 1b, 2b and 5.8% in Group 2a. In Group 1a there were one haemoperitoneum, one ischaemic colitis and one graft infection. In Group 1b there were 4 nefrectomies for renal carcinoma and three emergency hernia repairs within 18 months from AAA operation. In Group 2a the follow-up was uneventful. In Group 2b there was no acute complication of OD and 57.2% of patients were subsequently operated for OD. In the EVAR group the 30-day and late mortality rates were 0 and 25%, respectively and all deaths were cancer-related. Contemporary correction of OD in open surgery for AAA should be performed in clean wound cases, while clean-contaminated operations can be done only in selected cases. EVAR is a valid alternative technique to open vascular surgery for the concomitant treatment of aortic aneurysms and abdominal pathologies.

## Introduction

More and more frequently patients who have to undergo an aortic replacement due to an abdominal aortic aneurysm (AAA) are found to suffer from other abdominal pathologies. When choosing the therapy, it is therefore necessary to evaluate both risks and benefits of a combined treatment versus a treatment at two different times in consideration of the benign or malignant nature of the pathology associated with AAA; the risk of short term rupture must also be taken in consideration as far as the aortic lesion is concerned.

Two aspects seem to be particularly important: (1) the risk of infection of the aortic prosthesis, especially if the two pathologies are treated at the same time, but also if AAA is treated alone, when the associated pathology represents a potential septic focus; (2) the risk of a rapid evolution of AAA towards rupture, theoretically favoured by the biohumoral mechanisms generated by the laparotomic surgical trauma, if it is decided to treat the non-vascular pathology first.

To establish if the surgical timing strategy adopted until now is to be confirmed or if, otherwise, it needs to be modified, the present paper reports short- and long-term results, retrospectively recorded from a prospective database, on 315 patients suffering from non-vascular pathologies associated with AAA, compared with the results observed in 1,060 patients with AAA not suffering from associated pathologies who were operated on in the same period.

## Patients and methods

Out of 1,375 elective aortic replacements for AAA consecutively carried out at the Surgical Clinic of the Brescia University, with mean follow-up of 36 months, 315 cases showed other pathologies of surgical interest along with AAA. According to the published international guidelines [1, 2], these patients were subdivided as follows in consideration of the risk of infection connected with a surgical repair of their pathologies:

Group 1 (clean wound): constituted by 82 patients with associated pathologies allowing aseptic surgery (hernia, incisional hernia, renal carcinoma, adrenal neoplasms), 66 of which (80%) were treated at the same time (Group 1a) and 16 (20%) were not treated at the same time as aortic replacement (Group 1b) (Table 1).

**Table 1:** Group 1: pathologies allowing aseptic surgery

Associated pathologies	Total	Group 1a (%)	Group 1b (%)
Inguinal or inguinal-scrotal hernia	34	27 (79.4)	7 (20.6)
Incisional hernia	20	20 (100)	0
Umbilical hernia	11	11 (100)	0
Jatal hernia	6	1 (1.7)	5 (8.3)
Epigastric hernia	1	1 (100)	0
Renal carcinoma	7	6 (85.7)	1 (14.3)
Adrenal metastasis	1	0	1 (100)
Adrenal benign diseases	2	0	2 (100)
Total	82	66 (80)	16 (20)

Group 2 (clean-contaminated wound): constituted by 233 patients suffering from associated pathologies, the treatment of which would not allow to guarantee complete asepsis (cholelitiasis, ileum, colon and appendix benign and malignant pathologies, gastric carcinoma, prostate and bladder neoplasms, ovarian cyst), 86 of which (37%) were treated at the same time (Group 2a) and 147 (63%) were not treated at the same time as the aortic replacement (Group 2b) (Table 2).

**Table 2:** Group 2: potentially septic pathologies

Associated pathologies	Total	Group 2a (%)	Group 2b (%)
Cholelitiasis	107	57 (53)	50 (47)
Gallbladder adenomyosis	3	3 (100)	0
Diverticular disease	58	0	58 (100)
Meckel’s diverticulum	8	7 (87.5)	1 (12.5)
Appendix disorders	6	6 (100)	0
Benign haepatic pathology	5	0	5 (100)
Benign jejunoileal pathology	7	6 (86)	1 (14)
Ovarian cyst	1	1 (100)	0
Retroperitoneal angioma	1	1 (100)	0
Malignant haepatic neoplasia	7	3 (43)	4 (57)
Gastric carcinoma	4	2 (50)	2 (50)
Prostate carcinoma	15	0	15 (100)
Benign prostatic disease	3	0	3 (100)
Bladder carcinoma	8	0	8 (100)
Total	233	86 (37)	147 (63)

A total of 152 (Group 1a and Group 2a) patients underwent an aortic replacement and a contemporaneous repair of the associated pathology, whilst in 163 cases, only the aortic replacement was carried out. We considered a minimum interval of 30 days between the two interventions to define a treatment “staged”. The clinical and instrumental (abdominal US for all patients, annual CT for patients operated for neoplasia) follow-up was 36 months.

## Results

### Group 1: associated pathologies treatable with surgery not implying the risk of intraoperative contamination (clean wound)

Group 1a: Out of 66 cases surgically operated on at the same time for both pathologies, mortality was 0%. We found two (3.0%) complications after 30 days: one haemoperitoneum case, in a patient who had undergone an aortic replacement and a plastic surgery for umbilical hernia that required surgical revision for haemostasis and drainage; one ischaemic colitis, healed with medical therapy, in a patient who had undergone an aortic replacement and a plastic surgery for a left inguinal hernia. During follow-up, one case of infection was found in the third month (1.5%), in a patient subjected to aortic replacement and ventral hernia repair of periprosthesic abscess, only a surgical drainage was carried out. This was clinically successful and the patient had to periodically undergo CT for 3 years.

Group 1b: Three hernia repair interventions, one of which urgent and two of which elective, were necessary in the case of the 16 patients for whom it had been decided not to treat the associated pathology at the same time as the aortic replacement. These operations took place, respectively, 6, 12 and 18 months after the first surgical operation; out of the other 13 patients, 4 suffering from malignant [2] and benign [2] renal or adrenal tumours were all operated on 1 month after the first surgical operation, with lesions showing no evolution during the time after the first observation.

### Group 2: pathologies associated with surgical operations implying the risk of intraoperative contamination (clean-contaminated wound)

Group 2a: mortality in the 86 patients treated at the same time for both pathologies was 5.8% (four myocardial infarction and one multiple organ failure). Morbidity was to 6.2% (5/81). Mortality and morbidity only occurred in the 60 cases of cholecystectomy, none of which were in the complicated phase and with no evident association between cholecystectomy and negative events. No adverse event took place after associated surgical operations carried out for pathologies which appeared to be more risky. No further complications arose during follow-up in the 81 patients released (excluding the five deceased).

Group 2b: no mortality and morbidity was recorded in the 147 patients initially treated only for AAA. Surgical operations or endoscopic procedures were needed for 83 patients out of 145 (57.2%) in the months following the first operation. There were no complications and no evidence of the disease progression in oncologic cases: out of 50 patients known to suffer from cholelitiasis 12 were subjected to cholecystectomy, out of which two were urgent operations and 10 were elective; out of 15 patients suffering from prostatic neoplasia, 8 underwent prostatectomy, two endoscopic resection and five hormone therapy; patients harbouring bladder neoplasm underwent cystectomy in three cases and endoscopic treatment in five cases; one patient suffering from gastric neoplasia was subjected to gastrectomy 2 months after aortic replacement; out of the four patients suffering from hepatic neoplasia, two underwent chemoembolisation and two hepatic resection during the second month after the first operation; out of the 58 patients suffering from diverticular disease, 10 underwent colic resection between the 10th and the 14th month after the first operation.

A statistical comparison between the results of Group 1 and Group 2 considers patients operated on for both pathologies as well as patients operated on only for AAA lead to significant conclusions as far as mortality was concerned: the five patients operated on for both pathologies who died all belonged to Group 2a (*p* < 0.05 with respect to patients operated at the same time belonging to Group 1a). Results concerning morbidity were similar. A mortality rate of 3.25% and a morbidity of 4.6% were observed in the 152 patients of both groups operated on at the same time for both pathologies (Group 1a + 2a). This result was statistically similar to that of the 1,060 patients (mortality 2.1% and morbidity 4.2%) operated on only for aortic replacement and not suffering from associated pathologies and slightly higher than the result observed in patients suffering from both pathologies who had not undergone contemporaneous treatment (Group 1b + 2b).

As far as results during medium- and long-term follow-up are concerned, only one case of prosthetic infection was observed 3 months after the first surgical operation in a patient belonging to Group 1a. This indicates a total incidence of such complication of 0.6% in the 152 patients operated on at the same time (Group 1a + 2a). This complication was 0% in the patients suffering from other pathologies not treated at the same time (Group 1b + 2b): this difference is not statistically significant, neither it is significant when compared with the three cases (0.24%) observed in 1,060 patients operated on for AAA not suffering from other pathologies.

The total survival rate according to Kaplan–Meyer corresponded to 92% after 3 years and to 88% after 5 years for patients of Group 1a and patients of Group 2a operated on for both pathologies, and this result is statistically similar to the result obtained with the 1,060 patients (95 and 85%, respectively) who had undergone only aortic replacement and who did not suffer from associated pathologies.

EVAR was done in 12 patients, who had a infrarenal AAA of ≥4.0 cm (range 4.0–7.9 cm; mean size 5.5 cm) and concomitant abdominal disease or malignancy amenable for curative treatment. All aneurysms operated with <5 cm in diameter were saccular aneurysms with characteristics of “Impending rupture” (blister, blebs).

In the EVAR group, the AAA and abdominal disease or cancer were treated with one-stage procedure in six patients (one inguinal hernia, one linfoma, one splenic aneurysm, one cholelitiasis, two gastric carcinomas). The others six patients underwent a two-stage treatment (two renal carcinomas, one penis carcinoma, one linfoma, two rectum cancers) (in all cases with two different hospital admissions); in two, AAA was treated first. Patients in the EVAR group were followed up at 3, 6, 12 month and then every year. No patients died perioperatively. Postoperative complications (30-day and late morbidity) occurred in two patients (leak 1A developed 2 days after the operation, which required correction with ballooning; leak 2B revealed by 1 month TC after endovascular repair, with spontaneous resolution after 1 year). No sign of endograft infection was developed.

The 30-day and late mortality rates were 0 and 25%, respectively; all deaths were cancer-related.

## Discussion

The choice of the therapeutic sequence in patients suffering not only from aortic aneurysm but also from other abdominal pathology of surgical interest must be take into account the following general aspects which have a purely theoretical value [3, 4]: the risk of septic contamination in aortic prosthetic surgery must be reduced to a minimum in consideration of the very high morbidity and mortality associated with this complication. It is, therefore, very important to consider whether to treat the associated pathology by aseptic surgery or by surgery implying no septic risks [5, 6]. On the other hand, in the presence of neoplastic pathologies, it is important to consider that the surgical trauma deriving from the aortic replacement could have a negative impact on the immune system of the patient and therefore favour the progression and the diffusion of the non-treated neoplasia [7–13]. Furthermore, if an aortic replacement is carried out without treating the associated pathology, specific complications could arise from the latter. Finally, if it is decided to postpone the aortic replacement and to treat the associated pathology first, the laparotomic trauma could increase the risk of rupture of the abdominal aneurysm [9].

As far as abdominal wall pathologies and, in general, all potentially non-septic pathologies are concerned, our and other experiences [6–8] show that parietal plastic surgery does not increase the risk of complications and does not increase the rate of prosthetic superinfections. The paralytic ileum period and the gradual recovery of intestinal motility, always present after aortic replacement, can favour hernial complications, requiring a repeated urgent surgery, and therefore, cause possible infection foci. However, we postpone the parietal plastic surgery in 16 of our patients operated on for difficult aortic replacement requiring a prolonged anaesthesia.

In the case of neoplasia with a low septic risk, contemporaneous treatment seems to be even more appropriate as it avoids the risk of neoplastic progression or, vice versa, of AAA rupture. A typical example is nephrectomy for carcinoma: many Authors agree in proposing the concomitant treatment of AAA and renal carcinoma, because this procedure does not increase mortality and morbidity [3, 14–17], especially performing the nephrectomy in the first part of the operation [3, 16–18]. If partial nephrectomy is carried out, it seems, however, more prudent to carry out surgery at two different times, to reduce prosthesis contamination by possible urinary leak from the residual parenchymal. In our experience, the six nephrectomies and the upper polar resection carried out for neoplasia at the same time as aortic replacement did not lead to either immediate or long-term complications.

In case of associated potentially septic pathologies, the choice of the most appropriate timing is more complex, related to higher risks of aortic prosthesis infection. In our 86 patients in Group 2a, morbidity, including the risk of prosthesis infection, was not statistically different from patients in Group 1a or from the 1,060 patients operated only for AAA. However, mortality was completely different in this group: 5 patients out of 86 died (5.8%). This problem is relevant in patients with benign disease of the gallbladder, because biliary contamination represents a frightening cause of infection of the aortic prosthesis; we have also to consider the risk of acute cholecystitis after the aortic replacement. Several authors reported very high percentages of such septic complications which were particularly dangerous for the possible direct diffusion of the sepsis to the aortic prosthesis before its healing. Cadot et al. [19] report even an incidence of cholecystitis of up to 18% after the endovascular treatment of aneurysm. It is therefore probably appropriate to have an eclectic attitude in the choice of the therapeutic strategy. In our experience, we have not observed immediate and long-term septic complications. Other authors propose to proceed to cholecystectomy by video-assisted laparoscopy and then to carry out aortic replacement with an extraperitoneal approach, possibly by means of “gasless laparoscopy” to reduce the risk of acute evolution of AAA which could be favoured by the pneumoperitoneum [20, 21]. In our opinion, the laparoscopic approach has to be chosen for cholecystectomy in the case of surgery carried out at different times.

Frequently diverticular diseases of the colon not suspected of in the pre-operatory phase are found. In these cases, the choice is very clear: surgery cannot take place contemporaneously because of the elevate risk of direct endogenous contamination. If a complicated diverticulosis needing treatment is known before the aortic aneurysm diagnosis, the prosthetic replacement surgery must be carried out time after colic resection. In our experience one patient operated on for a large AAA suffering from diffused diverticular pathology ignored until the time of the operation showed a colon perforation in a very early stage (seventh day); he was subjected to urgent operation with an Hartmann’s procedure and had a regular post-operative course and he is healthy 44 months after operation.

In no case did we carry out an aortic replacement at the same time as the treatment of malignant bladder and prostate pathologies. In these cases the risks connected with the two associated pathologies are determinant to define the best timing for surgery which should be carried out at different times at a distance of at least 3–6 months.

The association of aortic aneurysm and malignant gastrointestinal neoplasia put a more complex choice concerning the surgical timing. As far as of colon cancer and gastric cancer are concerned, quite concordant opinions seem to be found the available literature [22, 23]: in the presence of abdominal aortic aneurysm with a high risk of impending rupture and asymptomatic colon neoplasia (not in an advanced stage and without the risk of short-term complications), it is recommended to treat the aortic pathology first; in the presence of abdominal aortic aneurysm with a medium diameter associated with an advanced stage colon neoplasia (with high risk of complications like intestinal occlusion or perforation in short time), it is recommended to treat colic pathology first [4, 24, 25]; in the presence of an abdominal aortic aneurysm with a high risk of rupture and of colon neoplasia in an advanced stage, the choice of the surgical “timing” certainly is more complex; in fact, some authors believe that a contemporaneous treatment of the two pathologies with one surgical operation [25] could be taken into consideration. We observed two cases of gastric neoplasia: one of these was in a very advanced stage and associated with an AAA with a diameter of >6 cm presenting a high risk of rupture in the short term. In this case, we decided to treat the two lesions at the same time. We believe, however, that this option should represent an absolute exception, even though in our specific case, we had a regular post-operative course without complications over the successive 36 months of follow-up.

These considerations appear to be quite obvious for “open” aortic surgery, but we think that they are valid also for endovascular aortic repair (EVAR); more cases of aortic endoprosthesis infection are reported in the literature [26–29]. In the last few years, several authors published their sporadic experience with the treatment of AAA through EVAR and of associated abdominal pathologies: eight cases of AAA associated with colon neoplasia operated on at two different times [30–33] and one case treated at the same time by endovascular exclusion of the aneurysm and successive colic resection. Veraldi and colleagues [34] report their experience both with open and endovascular treatment of abdominal aneurysm in association with colic resection for neoplasia and declare that they consider EVAR as an ideal treatment (possibly in one stage at the same time as the associated pathology) because of a lower risk of infection. In addition, Suffat and colleagues [35] report their experience with three cases of AAA associated with a malignant colic pathology and recommend EVAR in the first stage followed by colic resection a few days after the absence of relevant complications has been ascertained. We agree with Porcellini and Jesse [13, 36], reviewing its cases from 1997 to 2005, which consider EVAR as a valid alternative technique to open vascular surgery for the concomitant treatment of aneurysmatic and tumoral pathologies.

## Conclusions

In our experience, any combined surgery for the treatment of aneurysm abdominal aortic pathologies concomitant with other pathologies involves a higher surgical risk; therefore, it is important to assess the risk/benefit ratio of a contemporaneous versus double surgical treatment. Operation strategy and technique must be perfect to avoid surgical complications; since, in case of adverse outcomes, the surgeon would have to justify his aggressive choice.
